# Grasping the paradoxical nature of wisdom through unconscious integrative complexity

**DOI:** 10.3389/fpsyg.2022.1028951

**Published:** 2022-11-03

**Authors:** Christopher Kam, Christian R. Bellehumeur

**Affiliations:** School of Counselling, Psychotherapy and Spirituality, Faculty of Human Sciences, Saint Paul University, Ottawa, ON, Canada

**Keywords:** unconscious, integrative complexity, wisdom, paradox, psychoanalysis

## Abstract

There has been much progress in the scientific study of wisdom on both conceptual and empirical fronts in the past few decades. Despite all the progress being made, there are still gaps that can be filled to provide even more explanatory power and coherence. Although academic discourse on wisdom has included the ability to integrate issues in a complex manner, there is still room for improved theorizing on wisdom’s integrative complexity. Since integrative complexity has both conscious and unconscious dimensions, including the latter in discussions on wisdom will add a valuable aspect to its conceptualization. This article will argue how unconscious integrative complexity is the variable in wisdom’s conceptual equation that involves paradox, which is a well-known sign of wisdom. Explanations contrasting conscious integrative complexity and unconscious integrative complexity in reference to wisdom will be discussed. Then, the Archetypal Test of the Nine Elements will be proposed as a testing instrument to operationalize unconscious integrative complexity. After the conceptualization and operationalization are worked through, we will conclude with a couple examples to illustrate our reflections.

## Introduction

Since the beginning of the twenty-first century, scientific discourse on wisdom has gained momentum (Glück et al., 2013). Research has shown that wisdom is not only linked to wellbeing, but also overall health, life satisfaction, and resilience (Jeste and Lee, 2019). In addition, reasoning wisely has been associated with less negative affect, less depressive rumination, better social relationships, more positive compared to negative words in speaking, and longevity (Grossmann et al., 2013).

As much progress has been made in the study of wisdom and its effects on wellbeing, there is still progress to be made in its conceptualization, as there remains “controversy among wisdom researchers about the definition of wisdom” (Staudinger and Glück, 2011, p. 236). Part of the difficulty of defining wisdom is that it is a multidimensional concept (McKee and Barber, 1999) that is somewhat elusive. For instance, it is distinct from intelligence (Grossmann et al., 2013). Sternberg (1985) notes how wise individuals seem to be equal with intelligent people in problem solving and reasoning capabilities but also know when to listen to others, have flexibility in dealing with them, and consider both short and long term consequences. “Wise people probe inside “knowledge” to find its “deeper” meaning. They understand what they do and do not know, and the limits of what can be known” (McKee and Barber, 1999, p. 158). Wisdom seems to orient people toward a balance between positive and negative experiences that results in superior emotional regulation abilities rather than the sole pursuit of happiness (Grossmann, 2017; Grossmann et al., 2017). This is because wisdom seems to enable individuals to transcend a one-dimensional view of an issue with multidimensionality (McKee and Barber, 1999).

Despite the ongoing discussions of various perspectives on wisdom, one common agreement seems to be its multidimensionality. Jeste and Lee (2019) see wisdom as “a complex human trait with several specific components: social decision making, emotion regulation, prosocial behaviors, self-reflection, acceptance of uncertainty, decisiveness, and spirituality” (p. 127). Glück and Bluck (2013) define wisdom as a body of experience-based knowledge about fundamental life issues that is both broad and deep as well as explicit and implicit. Grossman et al. (2020) posit how wisdom considers different perspectives on issues as well as how to integrate them. Clayton and Birren (1980) see wisdom as integrating cognitive, reflective, and affective dimensions. Weststrate and Glück (2017) note that “wisdom is, and results from, the dynamic interaction of cognitive and non-cognitive resources” (p. 800). As these various scholars have conceptualized conscious and unconscious elements of wisdom, this same philosophy can be applied to conscious and unconscious dimensions of integrative complexity, which is a component of wisdom. There seems to be at least partial convergence and overlap between Weststrate and Glück’s (2017) dichotomy between cognitive and non-cognitive resources as well as Kam and Bellehumeur’s (2021) dichotomy between conscious and unconscious (also known as rational and non-rational) integrative complexity.

In this article we will make a case for the following view: in addition to conceptualizing wisdom with conscious integrative complexity, there is a need to account for unconscious integrative complexity, which the research on wisdom has a lack of. We will define these constructs from the overall developmental framework they come from, namely Adult Ego Development (AED), which has a history of conceptual interaction with wisdom. We will then argue why unconscious integrative complexity has a unique contribution to the ongoing conceptualization of wisdom, namely by providing a framework for the paradoxical dimension of it. We will then give a couple examples and end with some directions for future research.

## Integrative complexity’s relation to wisdom and adult ego development

Integrative complexity, also known as cognitive complexity (Fearon and Boyd-MacMillan, 2016), is the ability to differentiate among different dimensions of an issue and integrate the various aspects together (Conway et al., 2018). Since one characteristic of wisdom is the ability to integrate different elements of an issue together (Basseches, 1984; Sternberg, 1998; Yang, 2014; Grossmann, 2017; Grossman et al., 2020) while another involves effectively processing complexity (Glück and Bluck, 2013; Weststrate and Glück, 2017), the concept of integrative complexity seems appropriate to include in the scholarly discourse on wisdom. In addition to conceptual relevance, empirical research shows similarity in the benefits of integrative complexity and the benefits of wisdom. For example, integrative complexity helps resolve conflicts (Woodard et al., 2021) and helps people experience a more positive response to stress in situations that induce it, such as relational conflict (Fearon and Boyd-MacMillan, 2016). As adolescents grow in integrative complexity, they are better able to plan ahead, understand the long-term consequences of their behavior, show less negative behavior, resist peer pressure better, and appreciate the connections formed between emotion and behavior (Orr and Ingersoll, 1995). Integrative complexity also helps individuals understand issues better (Welfare and Borders, 2010) and have greater empathy (Heck and Davis, 1973).

Integrative Complexity is a component of Adult Ego Development (AED), which is the study of the development of the ego, also known as “the self,” in its process of maturation largely pioneered by Jane Loevinger (Loevinger, 1976, 1987; Singleton et al., 2021). Some key assumptions of AED are that psychological growth is characterized by paradigm shifts in seeing the world from simple to complex, static to dynamic, and inflexible to flexible (Cook-Greuter, 2004). Loevinger’s framework of AED explicitly aims to combine ideas on cognitive development and adult maturation (Staudinger and Glück, 2011; Hy and Loevinger, 2014). From this perspective, AED has relevance to wisdom since the maturity process of adulthood is frequently associated with wisdom (Bluck and Glück, 2005). Also, Loevinger (1976) received conceptual influence from Erikson (1968, 1984), who conceptualized wisdom as a personal maturation process later in life that dealt with the uncertainties of life by balancing seemingly opposite desires in a manner that transcends the limitations of the egoistic self (Brienza et al., 2018). It is worth noting that aging in and of itself does not necessarily grow AED, since factors such as a person’s level of openness and accommodative processing (the extent to which one reflects on difficult events and processes their transformative impact), have shown to play a significant role in AED (Lilgendahl et al., 2013). This trait of reflecting and processing significant life events has been associated with wisdom (Weststrate and Glück, 2017).

When AED happens, Loevinger argued that the ego/self consisted of undergoing a series of qualitatively adaptive shifts within a path of hierarchically organized stages of meaning making. Here, each stage is more maturely evolved than the previous, as there are adaptive transformations on a fundamental level of four interconnected dimensions of the personality: integrative complexity, interpersonal relationships, impulse control, and conscious preoccupations. The first two dimensions, integrative complexity and interpersonal relationships, are especially relevant to wisdom since wisdom is associated with integrating ideas in one’s context (Sternberg, 1985), integrating deeper insight into generally known facts (Ardelt, 1997, 2003), integrating different opinions and perspectives (Yang, 2014), confronting the complexities of life (Glück and Bluck, 2013), better social relationships and interpersonal wellbeing (Grossmann et al., 2013), empathic and benevolent perspective taking (Ardelt, 1997, 2003), and prosocial behavior (Jeste and Lee, 2019). Although integrative complexity’s relevance to wisdom involves predominantly the first two dimensions listed here, a premise of Loevinger’s framework is that these 4 dimensions of the maturing self in AED are inseparably interconnected. Integrative complexity and interpersonal relationships are important here, as AED sees wisdom as advanced psychological maturity in adulthood that includes prosocial motives and dialectical reasoning (Hy and Loevinger, 2014). There is some empirical support for these arguments, as more exploratory forms of reflective processing tend to be connected with higher ego development (Lilgendahl and McAdams, 2011) and are also positively associated with wisdom (Weststrate and Glück, 2017).

## Conscious and unconscious integrative complexity

The complexity of the human mind has been documented in recent years with respect to both conscious and unconscious capacities for sophistication. By “unconscious” process, we refer to Boag’s (2017) definition, where he notes that “a mental process is descriptively unconscious if we are presently unaware of it. For example, a belief would be described as descriptively unconscious if it was believed, without the person currently being aware of having the belief” (Boag, 2017, p. 2). For example, master chess players unconsciously recognize many meaningful patterns in chess, which novice players can miss, and can often make their next move with a quick glance on the chessboard (Myers and Twenge, 2019). Kruglanski and Gigerenzer (2011) note that “Heuristics that are less effortful and in which parts of the information are ignored can be more accurate than cognitive strategies that have more information and computation” (p. 97).

With respect to formal theories that have some empirical support for such assertions, Unconscious Thought Theory (UTT) has shown some evidence for the superiority of unconscious decision making (Bargh et al., 2012). UTT argues that after a period of conscious thought on information relevant to an issue, a period of unconscious deliberation (where conscious thought is directed elsewhere) produces better quality judgments, provided that in the beginning there was a conscious intention to make the best decision. For example, unconscious thought (activated in experiments where participants are distracted for a few minutes from a complex decision only to return to make a judgment afterward) was shown to lead to superior judgments in legal justice cases compared to participants who made immediate conscious judgments (Ham et al., 2009).

Furthermore, conscious thought is smaller in attentional capacity but seems superior at rules based processing with precision whereas unconscious thought seems superior in breadth of processing capacity, is more intuitive without being led by rules-based processing [although it can passively conform to it (Lewicki et al., 1992)], can better weigh the relative importance of the aggregate aspects of issues, and gravitates toward divergence and creativity (Dijksterhuis and Nordgren, 2006; Nordgren, 2011; Vieira et al., 2017). Nordgren (2011) argued that a combination of conscious and unconscious processes solves complex problems better than either itself. Lastly, Baumeister and Masicampo (2010) concluded from a social psychology perspective that the main triggers for social behavior are unconscious, but conscious processes also play an important role as well since they are capable of redirecting unconscious behavior or judgmental impulse.

With respect to Loevinger, she acknowledged the reality of the dynamic unconscious and how AED has both cognitive and non-cognitive elements to it (Loevinger, 1976, 1993). Furthermore, Loevinger’s AED framework is also consistent with some psychoanalytic premises, which Loevinger (1993) herself said were compatible with her theorizing. It has also been suggested that Loevinger’s AED theory is consistent with Jung’s (1921) theorizing of individuation, specifically in five areas: individuality, self-awareness, wholeness, autonomy, and complexity (Broughton and Zahaykevich, 1988). Jung’s psychoanalytic theory, by its very nature, deals with the unconscious and how it seeks to integrate seemingly opposite unconscious desires in the process of development in individuation.

Taking into account cognitive and non-cognitive dimensions of wisdom (Glück and Bluck, 2013; Weststrate and Glück, 2017) that have partial overlapping with Loevinger’s (1993) theorizing of cognitive and non-cognitive elements of AED as well as the compatibility of her framework with psychoanalysis, there is a need to define unconscious integrative complexity, operationalize it, and conceptually integrate it within the overall scholarly discourse on wisdom. When defining the construct of unconscious integrative complexity, we take into account the unconscious dimension of Medvene et al.’s (2006) notion of interpersonal cognitive complexity, which is “the ability to perceive others in relatively complex and personalized terms” (p. 220) as well as “the number of psychological constructs that people use to describe others” (p. 220–221). Integrating these ideas together, unconscious integrative complexity (which can also be termed unconscious interpersonal complexity in matters pertaining to social aspects of wisdom) can be defined as the degree of complexity in the number of unconscious psychological constructs involved in experiencing life, particularly with regard to matters of wisdom such as relationships (Kam and Bellehumeur, 2021). As we have argued for the conceptual soundness of the unconscious dimension of integrative complexity in wisdom, there is a need to offer an empirical method to measure this construct.

Based on the seminal work of Durand (1999)’s Anthropological Structures of the Imaginary (ASI) (original book, 1960), there is a projective test, called the AT.9 (Archetypal Test with 9 elements), that was developed by Durand (2005). This test empirically supports and operationalizes, Durand’s (1999) main categories of the unconscious source of a person’s imagination: heroic and mystical (which are more one-dimensional), and synthetic categories (which are more qualitatively multidimensional with the co-existence of opposite themes dynamically coexisting). Within the synthetic category of the imaginary, Durand (1999) directly refers to the acknowledgments of the Yin-Yang dialectics, which is part of mature happiness proposed by Wong and Bowers (2018).

As the AT.9 takes into account paradox, it is well suited to assess the degree of a person’s unconscious symbolic capacity to embrace the co-existence of opposites. Many conceptual writings and empirical studies have demonstrated how the AT.9 not only embraces paradox, but can assess the unconscious (non-rational) dimension of one’s imagination, worldview and symbolical capacity. For instance, the relevance of the ASI and/or AT.9 have been conceptually and empirically demonstrated in the context of resilience and spirituality (Bellehumeur, 2011; Nguyen et al., 2018; Bellehumeur and Carignan, 2021; Yeung and Bellehumeur, 2021), wellbeing, virtues, as well as personal strengths (Bellehumeur et al., 2017). The conceptual and empirical adequacy of the ASI and the AT.9 have also been shown in the context of the paradoxical dimension of boundaries in psychotherapy, theology, and anthropology (Bellehumeur and Chambers, 2017; Bellehumeur, 2020; Kam and Bellehumeur, 2020a,b); along with various concepts associated with interpersonal styles as well as and marital, family, and psychotherapeutic relationships (Bellehumeur et al., 2013; Bellehumeur, 2014a,b; Bellehumeur and Carignan, 2018). The relational dimension of the AT.9 has also been demonstrated through its use in studies measuring how collaborative someone is with others (Bellehumeur et al., 2013), and operationalizing how bicultural individuals navigate relationships while living in two cultures (Yeung, 2018).

In short, we propose that the AT.9 can be utilized to measure the level of sophistication of integrative and interpersonal experience in the unconscious dimensions of a person’s mind since “[the AT.9] attempts to measure the non-rational dimensions of a person’s interpersonal complexity” (Kam and Bellehumeur, 2021, p. 176). As a projective test, it assesses unconscious content (Cervone and Pervin, 2019) by taking into account symbolic and archetypal material, both of which involve aspects of one’s unconscious (Cook-Greuter, 1999; Schlamm, 2014). From these qualities, the AT.9 can help researchers operationalize a person’s non-rational capacity toward complexity since the narrative patterns, mythopoetic elements, and degree of multi-layered paradoxical organization in a person’s unconscious imagination is assessed. Since the AT.9 measures intuitive, non-rational, and symbolic dimensions of the psyche, it is particularly fitting for operationalizing unconscious integrative complexity since these are key elements of it (Cook-Greuter, 1999; Kam and Bellehumeur, 2021).

## The conceptual overlap of unconscious integrative complexity and underlying dimensions of wisdom

The conceptualization and operationalization of unconscious integrative complexity involving the intuitive, non-rational, and symbolic dimensions of the human psyche is consistent with a historical take on the study of the unconscious, which recognizes a mythopoetic function of it (Ellenberger, 1970). It is also consistent with empirical work with Short-Term Dynamic Psychotherapy where lifting defense mechanisms can result in unconscious imagery emerging that symbolizes deeper emotions behind the conscious defenses (Davanloo, 1987; Abbass et al., 2012; Town et al., 2013; Johansson et al., 2014). This is similar to how wisdom, in its very nature, requires a person to explore their negative emotions openly in a way that bypasses their defense mechanisms (Glück and Bluck, 2013; Weststrate and Glück, 2017; Glück et al., 2019). As unconscious integrative complexity is intuitive, non-cognitive, and symbolic while wisdom itself has aspects that are intuitive (Clayton and Birren, 1980), non-cognitive (Glück and Bluck, 2013; Weststrate and Glück, 2017), and metaphorical/poetic (Zwikstra, 2008), there is overlap in the type of psychic texture that both share. This type of psychological fabric is different from its cognitively logical counterpart and instead has a qualitatively different texture that is open to paradox, which is an aspect of wisdom (Clayton, 1975, 1983).

Advanced levels of unconscious integrative complexity have a mature depth on life matters with a paradoxical nature that is comfortable with the ambiguity of opposites coexisting in healthy tension (Kam and Bellehumeur, 2021). This is consistent with Sternberg’s (1990) take on wisdom not seeking to eliminate ambiguity, but being tolerant of it. Wisdom has been known to be characterized by the paradoxes of human existence that comes from the journey of transcending opposites (van Deurzen, 2014; Wong and Bowers, 2018). Here, the intuitive and non-rational parts of the complexity become integrated and can be captured through symbolic expression (Cook-Greuter, 1999). As wisdom involves prudence in dealing with ill-defined problems (Grossman et al., 2020) with some engagement in non-cognitive resources (Glück and Bluck, 2013; Weststrate and Glück, 2017), the type of paradox required here is one that provides non-structural coherence in seemingly opposite principles simultaneously coexisting. With this in mind, wisdom finds mysterious coherence in paradoxes (Tickerhoof, 2002), where the conscious mind does not fully understand how a paradox lacks contradiction while the paradox itself has an experiential, non-linear consistency to it. For example, while the cognitive mind can be open to the complexity of how the passage of time can occur both slowly (e.g., the days) and quickly (e.g., the years/decades) in the same life, it has trouble expressing how this is the case through pure rational logic. Here, unconscious integrative complexity can integrate the non-linear, intuitive and non-rational parts of the complexity in a paradoxical way that is somehow experientially consistent and express it with poetic symbolism (e.g., “When I’m bored, the minute hand of the clock seems to conspire with the universe in ticking slower. But when I’m animated in the flow of my favorite activity, time moves both fast and slow like, the Flash, in slow-mo”).

## Examples

This article will close with a couple of examples. Some individuals may recognize that they are so called “control freaks” who obsess about controlling events to acquire perfect security and a sense of “peace” for matters they care about. This can turn into an obsession where the preoccupation of controlling events for perfect security and “peace” can make it hard to surrender what is out of one’s control. Here, surrendering control would lead to an actual sense of peace. Through reflection on the level of conscious integrative complexity, one can realize the contradiction of the obsession of control as a means towards “peace” with the actual peace that comes with surrender and recognize that they cannot coexist. However, through extended holistic reflection involving unconscious integrative complexity, this contradiction can qualitatively transform into a higher order paradox. Here, one realizes: “Wanting perfect security in events creates pressure for perfect peace. Accepting imperfect security in events releases pressure for perfect peace.” Ironically, the former sabotages authentic peace while the latter facilitates it.

For other individuals there is a longing for perfect love with other humans with a perfect connection. This can turn into an obsession where the preoccupation for perfect love and connection with others can make it hard to be content with the imperfections of human relationships. Through reflection on the level of conscious integrative complexity, one can realize the conflict of the obsession with perfect love/connection with the peace of contentment with imperfect human relationships and recognize that they cannot coexist. However, through extended holistic reflection involving unconscious integrative complexity, this contradiction can qualitatively transform into a higher order paradox. Here, one realizes: “Wanting perfect love in humans creates pressure for perfect connection. Accepting imperfect love in humans releases pressure for perfect connection.” Ironically, the former sabotages authentic love in relationships while the latter facilitates it.

The latter realizations involve knowing one’s human limitations (Taranto, 1989), which is an aspect of wisdom. They also open up deeper and more profound understandings of knowledge (Kekes, 1983; Chandler and Holliday, 1990; Sternberg, 1990; Ardelt, 1997, 2003). Furthermore, these higher ordered realizations allow for paradoxes that bring a sense of peace in dealing with uncertainty, which is also a characteristic of wisdom (Meacham, 1990; Brugman, 2006; Grossmann et al., 2010, 2013). These qualities of awakening that occur in the universe of paradox transcend the psychic laws in the universe of conscious cognition with its logical calculations of structural coherence and linearly calculated contradictions. Qualitative shifts in unconscious integrative complexity create breakthroughs in the egoistic encounter with life, unlocking the mental handcuffs of one-dimensionality into the freedom of soulful multidimensional paradox.

## Future directions for research

There has been much advancement in conceptualizing wisdom from a scientific vantage point within the past few decades. However, since wisdom in its nature seems to continually have aspects of it that are elusive, there will always be room for new but relevant factors to be incorporated. Unconscious integrative complexity seems to fill a meaningful gap in the research since past assumptions in the literature seem to extend a welcoming spot for it. Aside from more conceptual fine-tuning, there is also a need for empirical research on unconscious integrative complexity in relation to wisdom. Since this is unexplored territory for both conceptual and empirical research, there is room for the use of various methodologies. For example, In addition to qualitative interviewing, there is room for the combined use of various quantitative instruments such as the Washington University Sentence Completion Test (Hy and Loevinger, 2014) and the AT.9 which can set the direction for future research. The former test can assess integrative complexity’s more conscious dimensions while the latter can assess integrative complexity’s more unconscious ones. Such research will better help humanity understand more about the multifaceted nature of wisdom, particularly its dimensions hidden beneath consciousness, and how to best acquire it.

## Author contributions

CK was the primary author of the initial version of this manuscript. CB helped make revisions to the manuscript to make it stronger and provided about 25 additional references to better support the relevance of using an empirical test to assess the unconscious. Both authors contributed to the article and approved the submitted version.
